# Autologous CIK Cell Immunotherapy in Patients with Renal Cell Carcinoma after Radical Nephrectomy

**DOI:** 10.1155/2013/195691

**Published:** 2013-12-09

**Authors:** Yajing Zhang, Jin Wang, Yao Wang, Xue-Chun Lu, Hui Fan, Yang Liu, Yan Zhang, Kai-Chao Feng, Wen-Ying Zhang, Mei-Xia Chen, Xiaobing Fu, Wei-Dong Han

**Affiliations:** ^1^Biotherapeutic Department, Chinese PLA General Hospital, Beijing 100853, China; ^2^Department of Cadre Health Care, Naval General Hospital, Beijing 100048, China

## Abstract

*Objective*. To evaluate the efficacy of autologous cytokine-induced killer (CIK) cells in patients with renal cell carcinoma (RCC). *Methods*. 20 patients diagnosed with TNM stage I or II RCC were randomly divided into two groups, a CIK cell treatment group and a control group. The endpoint was progression-free survival (PFS) evaluated by Kaplan-Meier analyses. *Results*. CD3^+^, CD3^+^/CD8^+^, CD3^+^/CD4^+^, and CD3^+^/CD56^+^ levels increased after CIK cell culture (*P* < 0.01). The median PFS in CIK cell treatment group was significantly longer than that in control group (PFS, 32.2 months versus 21.6 months; log-rank, *P* = 0.032), all patients were alive during the course of followup, and there are no statistically significant differences between two groups in OS (log-rank, *P* = 0.214). Grade III or greater adverse events were not observed. *Conclusions*. CIK cells treatment could prolong survival in patients with RCC after radical nephrectomy and showed acceptable curative effect with potential enhancement of cellular immune function. This trial is registered with Clinicaltrials.gov NCT01799083.

## 1. Introduction

Renal cell carcinoma (RCC), a human kidney cancer from the proximal tubular epithelium, accounts for approximately 3% of adult malignancies [1]. Improvements in radiological evaluation have enabled the incidental detection of more than 50% of renal cancers at an early stage [2]. Traditional treatment modalities such as chemo- and radiotherapy have shown overall response rates of 2%–6% [3, 4]. The limited success of these treatments indicates that further efforts are needed to improve the current therapeutic modalities and to explore novel therapies for RCCs to improve patient care and increase survival [5, 6]. Immunotherapy has recently become the fourth major modality for the treatment of malignant tumors after surgery, radiotherapy, and chemotherapy [7–9]. In the last few years, cytokine-induced killer (CIK) cells have been recognized as a novel type of antitumor effector cells, and their application has evolved from experimental observations into early clinical studies. CIK cells show a high proliferation rate and cytotoxic activity in vitro, with stronger antitumor activity and a broader spectrum of targeted tumors than other reported antitumor effector cells [8, 10]. Furthermore, CIK cells can regulate and generally enhance immune function with feasibility and low toxicity in patients with cancer [10]. The purpose of the present study was to evaluate the clinical efficacy of CIK cell immunotherapy in patients with early renal cell carcinoma after radical nephrectomy.

## 2. Materials and Methods

### 2.1. Patient Eligibility

The study was approved by the Institutional Review Board (IRB) of the General Hospital of the People's Liberation Army, and all patients signed a consent form for participation in the study in compliance with the *Declaration of Helsinki*. Patients with RCC and pathologically confirmed clear cell carcinoma were eligible for participation in the study. Patient eligibility included the following criteria: granulocyte count ≥3.5 × 10^9^/L; hemoglobin level ≥100 g/L; platelet count ≥100 × 10^9^/L; bilirubin and creatinine equal to or less than the institutional normal limits; life expectancy ≥12 weeks; measurable or evaluable disease; no immunotherapy, chemotherapy, or radiotherapy within 4 weeks (washout for 4 weeks); and negative serological tests for hepatitis B, hepatitis C, and HIV. Patients with serious illness or an active secondary malignancy were excluded. All patients were informed of the investigational nature of the study and signed informed consent in accordance with institutional guidelines. Each patient underwent a complete pretreatment clinical evaluation, including clinical history, physical examination with assessment of performance status, laboratory studies, and analysis of radiographic studies.

### 2.2. Patient Demographics

A total of 20 patients (17 men and 3 women) with unilateral, locally advanced (TNM stage I or II) RCC who had undergone radical nephrectomy of the primary tumor were recruited into the present study at the General Hospital of the People's Liberation Army between January 2009 and April 2010 and randomly assigned to control and CIK cell treatment groups. No statistically significant differences in age, sex, physical condition, and Motzer Criteria Factors [11] (Karnofsky performance status, corrected calcium, LDH level, hemoglobin level, and time from diagnosis to systemic radical nephrectomy) were observed between two groups. Patients were diagnosed according to the International Union against Cancer (2002) staging classification [12]. The CIK cells treatment group included 10 patients, 9 men and 1 woman, with a mean age of 58.2 years (range, 43–79 years). Six patients were diagnosed with left RCC and four with right RCC. The average size of tumors was 3 cm × 2.5 cm × 2.7 cm. The control group included 10 patients, 8 men and 2 women, with a mean age of 57 years (range, 49–74 years). Five patients were diagnosed with left RCC and five with right RCC. The average size of tumors was 3.2 × 2.5 × 2.4 cm. Clinical, pathological, and Motzer Criteriae Factors characteristics of patients are summarized and detailed in Tables 1(a), 1(b), and 1(c); besides, there are no statistically significant differences between two groups in comparison of Motzer Criteriae Factors (Karnofsky performance status (KPS), corrected calcium, LDH level, hemoglobin level, and time from diagnosis to systemic radical nephrectomy) (Table 1(c)).

### 2.3. Reagents and Apparatus

All reagents met the clinical criteria. Serum free medium was from Gibco (Carlsbad, CA, USA); recombinant human interferon (rhIFN-g) and recombinant human interleukin-2 (rhIL-2) were from PeproTech (Rocky Hill, NJ, USA). Anti-CD3 monoclonal antibody was obtained from Pharmingen (San Diego, CA, USA). Thymopentin for injection was purchased from Beijing Shuanglu Pharmaceutical Co. Ltd. (Beijing, China) and antibodies for T lymphocyte subsets were from BD (Franklin Lakes, NJ, USA). The FACS-420 flow cytometer was from Becton-Dickinson FACS Systems (Sunnyvale, CA, USA), and data analysis was performed with CellFit software (Becton-Dickinson Inc., San Jose, CA, USA).

### 2.4. Preparation of Cytokine-Induced Killer Cells

All the technicians for CIK cell culture and quality control were healthy and received training in good manufacturing practices. Informed consent was obtained from all patients prior to the study. A total of 54 mL of venous blood was obtained in the morning under fasting conditions, and peripheral blood mononuclear cells (PBMCs) were subsequently isolated. The PBMCs were grown in serum free medium and cell density was adjusted to meet predetermined criteria; the growth medium was supplemented with rhIFN-*γ* (final concentration of 2000 U/mL). The cells were maintained in gas-permeable cell culture bags at 37°C and 5% CO_2_. On the following day, rhIL-2 and CD3 McAb were added to a final concentration of 1000 U/mL and 50 ng/mL, respectively. On day 0 of culture, 1000 U/mL recombinant human interferon-(IFN-) *γ* (Peprotech, New Jersey, USA) and 1000 U/mL recombinant human interleukin-2 (rhIL-2; Peprotech) were added to the culture medium. The cells were cultured in a humidified 5% CO_2_ incubator at 37°C. Fresh GT-T551 medium with 1000 U/mL rhIL-2 was added every 3 days. After about 14 days of culture, the CIK cells had to meet the following criteria prior to transfusion: the proportions of CD3^+^, CD8^+^ and CD3^+^/CD56^+^ cells were >90%, >65%, and ≥20%, respectively, and cell viability, detected using trypan blue staining, was >95%. Approximately 2~10 × 10^9^ CIK cells were harvested per flask, with a survival rate of >95%.

### 2.5. Antibodies and Flow Cytometric Analysis

The following antihuman antibodies were used to stain cell surface markers to establish the CIK phenotype: CD4-fluorescein isothiocyanate (FITC), CD8-phycoerythrin (PE), CD3-chlorophyll protein complex (PerCP), and CD56-allophycocyanin (APC). The antibodies and isotype-matched monoclonal antibodies were purchased from BD Biosciences (California, USA). Data acquisition was performed using a FACSCalibur flow cytometer (BD Biosciences).

### 2.6. Treatment Regimen of Cytokine-Induced Killer Cells

**Figure 1:**
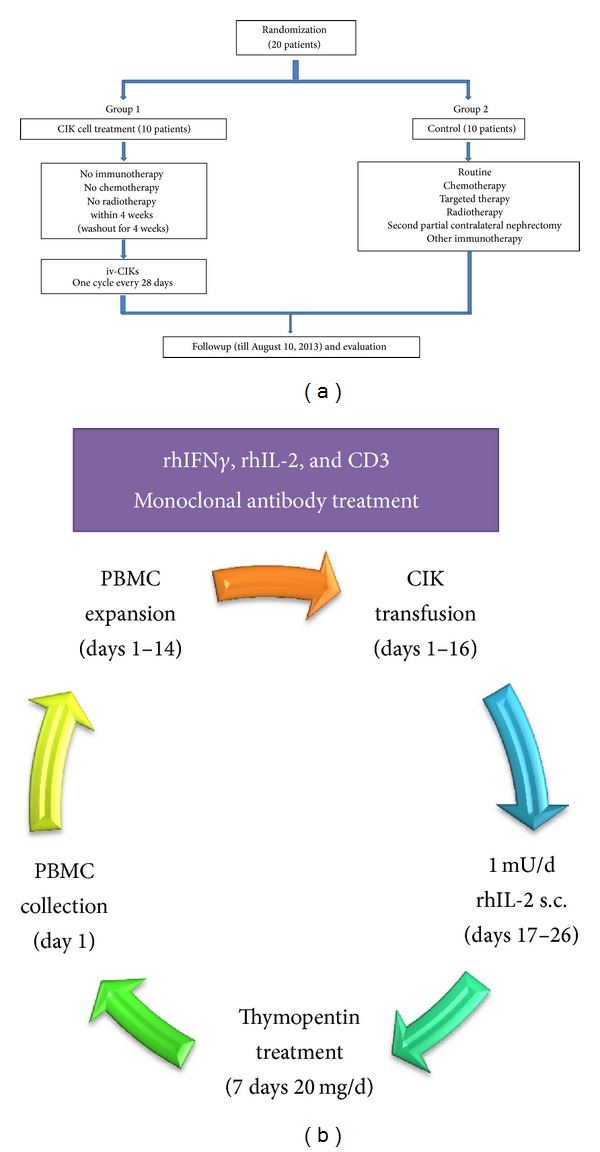
(a) Trials and treatments of the two groups sectionalization. (b) Treatment protocol: cytokine-induced killer (CIK) cell transfusion cycle. Peripheral blood mononuclear cells (PBMCs) were cultured for 14 days in the presence of recombinant human interferon gamma (rhIFN-*γ*), recombinant human interleukin-2 (rhIL-2), and anti-CD3 monoclonal antibody before transfusion for two consecutive days. Patients were injected with rhIL-2 subcutaneously at 1 mU/day for 10 days immediately after transfusion for 10 days. Thymopentin was injected intramuscularly for 7 days before the next PBMC collection and culturing.

The patients received thymopentin (20 mg/day) via intramuscular injection 1 week before PBMC collection for 7 consecutive days. After PBMC collection, thymopentin (20 mg) was injected intramuscularly three times per week until 1 week before the next cycle (Figure 1). After CIK cell transfusion, patients were injected subcutaneously with 1 mU rhIL-2 each day for 10 days (from day 17 to day 26). CIK cell transfusion (1~5 × 10^9^ CIK cells per infusion and 2~10 × 10^9^ CIK cells infusions totally) was performed and transfused back to the patients for two consecutive days intravenously during one course of treatment. Two weeks after the final transfusion, blood was collected, and CIK cells were harvested. The patients participating in this study did not receive any other treatment during CIK cell therapy.

### 2.7. Clinical Examinations and Assessment

The patients were followed up until they were lost to followup, died or until the end of followup on August 10, 2013. Patient followup was the same for the immunotherapy and control groups, and was performed every 3 months for the first 2 years after CIK cell therapy, every 6 months for the next 2 years, and yearly thereafter. Clinical and laboratory tests were performed at each visit. The main parameters were as follows: (i) general condition and physical examination, with signs and symptoms were assessed before and after treatment; (ii) serum tumor markers; (iii) routine blood tests for liver and kidney function were performed every 2 weeks during the treatment; (iv) cellular immune response was assessed by detection of peripheral lymphocyte subsets before and after treatment (CD3^+^, CD8^+^, CD3^+^/CD8^+^, CD3^+^/CD4^+^, and CD3^+^/CD56^+^); (v) imaging studies included ultrasonography performed every 3 months to detect abdominal and superficial lymph nodes, chest and abdominal computed tomography (CT) and/or magnetic resonance imaging (MRI) every 6 months, and whole-body positron emission tomography (PET)/CT once per year; (vi) Zubrod-ECOG-(eastern cooperative oncology group-) WHO scores were determined according to the Karnofsky performance status (KPS) scale [13] and survival time (from the end of CIK therapy to the time of survey) was recorded; (vii) objective tumor response was assessed every 2 months using the Response Evaluation Criteria in Solid Tumors (RECIST) method and reported as complete response (CR), no change (NC), partial response (PR), stable disease (SD), and progressive disease (PD).

### 2.8. Statistical Analysis

Statistical analysis was performed using SPSS 21.0 software (SPSS Inc., Chicago, IL, USA). The quantitative data were presented as $\overset{-}{\chi }\pm S$, and a *t*-test was used to compare the means between two groups. A value of *P* < 0.05 was considered to be statistically significant.

## 3. Results

### 3.1. Quality Control in Cell Culture

Cell cultures were routinely evaluated for the presence of bacteria, fungi, and mycoplasma by the Department of Microbiology and our laboratory. Cells testing negative for all bacteria, fungi, and mycoplasma were defined as negative. All the cells used for transfusion were negative for these microorganisms, which ensured the safety of treatment.

### 3.2. Phenotype Changes

**Figure 2:**
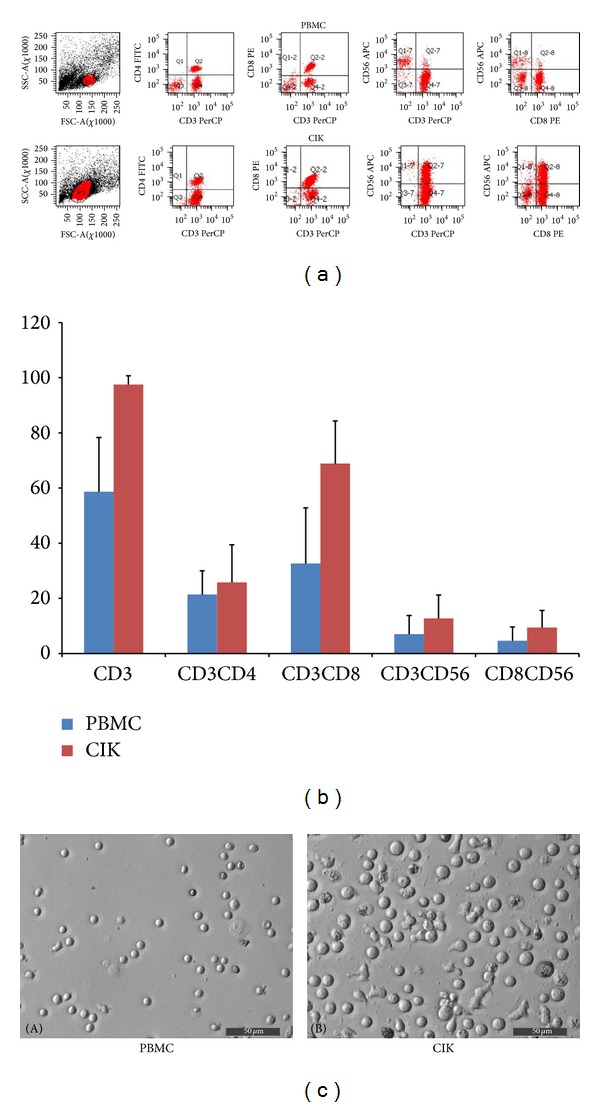
Phenotype analysis of cells from patients and detection of CIK cells and leukemic markers by FACS analysis. All cell samples for phenotype analysis were stained with FITC-conjugated antibodies against CD4, PE-conjugated antibodies against CD8, and APC-conjugated antibodies against CD56. (a) Typical phenotype analysis of PBMCs and CIK cells from CIK cell treatment group patient 7. (b) Comparison of the phenotype analyses of PBMCs and CIK cells. Phenotype comparisons were performed in samples from 10 patients who received CIK cell treatment, and the results were expressed as means ± SD. (c) Lymphocyte culture (PBMC and CIK). (A) PBMCs before isolation, induction and culture; some T lymphocytes can be seen in the peripheral blood. (B) After isolation, induction, and culture, lymphocytes become larger.

The average culture duration for peripheral blood lymphocytes was 13.39 ± 1.6 days. The average number of mature lymphocytes was (3.6 ± 0.77) × 10^9^ cells, and the average fold change of amplification was 463 ± 156.86. The survival rate of these cells was 97.681 ± 1.41%. Cells were analyzed by flow cytometry immediately after blood collection and again after 13 days of culture. Analysis of phenotypes showed a significant increase in the proportion of CD3^+^, CD8^+^, CD3^+^/CD8^+^, and CD3^+^/CD56^+^ T lymphocytes and a slight decrease in the number of CD3^+^/CD4^+^ T lymphocytes (Figure 2, Table 2).

### 3.3. Changes in Lymphocyte Subsets

Reexamination of peripheral lymphocyte subsets at 6−8 days and 12−14 days after CIK cell transfusion showed a dramatic increase in the proportion of CD3^+^, CD3^+^/CD8^+^, and CD3^+^/CD56^+^ cells (Table 3).

### 3.4. Adverse Events of Autologous Cytokine-Induced Killer Cell Transfusion

No significant changes in vital signs and no instances of rash, digestive discomfort, anaphylactoid reaction, tumor lysis syndrome, or headache were detected. Mild arthralgia, laryngeal edema, fatigue, and low-grade fever were noted in three patients during the course of lymphocyte infusion or during the early stages of rhIL-2 treatment. Adverse events of grade III or greater were not observed in any patient. All adverse events were resolved and disappeared without intervention within 24 h or were treated by symptomatic treatments such as antiallergy medicines (Table 4).

### 3.5. Treatment Response

All patients were alive during the course of followup. The general condition of patients was significantly improved after two courses of CIK cell transfusion including decreased malaise, improved mental state, increased food intake, and alleviation of cancer-related pain. The median follow-up period was 44 months; six patients (60%) in the CIK cell treatment group achieved a complete response, two patients (20%) had a partial remission, and two patients showed stable disease after CIK cell treatment, with an overall objective response rate of 80%. By the end of followup, two PR patients showed disease stabilization. In the control group, there were five complete responders (50%), with an overall objective response rate of 50%. Three patients (30%) had disease stabilization, and in two patients (20%), continuous disease progression was observed despite therapy.

### 3.6. Progression-Free Survival and Overall Survival

**Figure 3:**
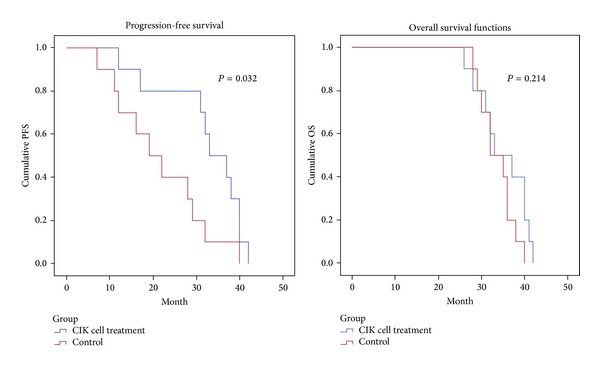
Kaplan-Meier estimates of progression-free survival (PFS) and overall survival (OS). Left figure: PFS. *Blue line:* CIK cell treatment group. *Red line:* control group. Log-rank: *P* = 0.032. Right figure: OS. *Blue line:* CIK cell treatment group. *Red line:* control group. Log-rank: *P* = 0.214; all patients were alive during the course of followup, and there were no statistically significant differences between two groups in OS.

The progression-free survival (PFS) and overall survival (OS) of each patient are described in Tables 1(a) and 1(b). The average PFS and OS in the CIK cell treatment group were 32.2 months and 35 months and those in the control group were 21.6 months and 33.6 months. PFS and OS curves in the CIK cell treatment and control groups are shown in Figure 3, which shows that the patients in the CIK treatment group had a significantly better PFS than those in the control group (log-rank, *P* = 0.032); all patients were alive during the course of followup, and there are no statistically significant differences between two groups in OS (log-rank, *P* = 0.214).

### 3.7. Imaging Features

**Figure 4:**
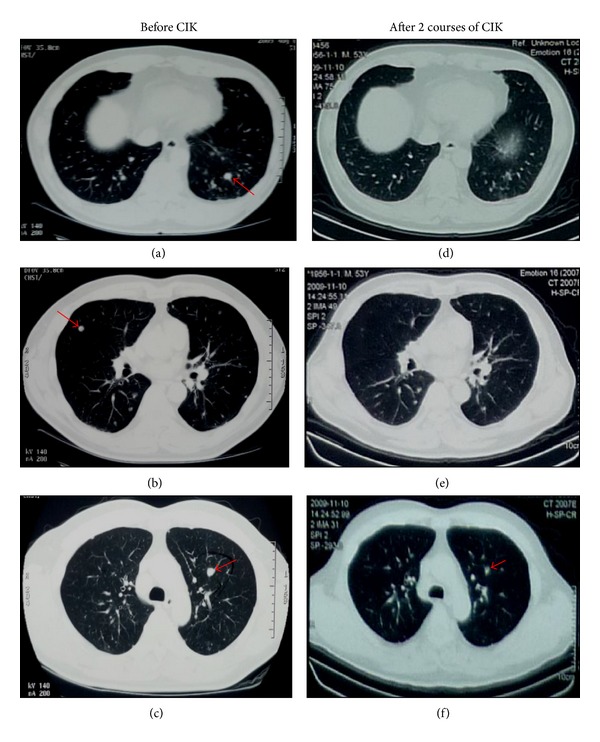
Example of chest CT before and after CIK cell treatment. ((a)–(c)) Images from Unique Patient Number (UPN) 7, who was diagnosed with pulmonary metastasis after radical nephrectomy and before CIK cell treatment. ((d)–(f)) Images from the same patient after two courses of CIK cell treatment. (a) A solid space-occupying lesion (0.9 × 1.2 cm) was observed in the left lung. (b) A circular lesion (0.5 × 0.5 cm) with high density and clear boundary was observed in the right lung. The CT value was 22 Hu in the plain scan. (c) The image shows a space-occupying lesion (1.1 × 0.9 cm) with a high density signal and a clear boundary. (d) After two courses of CIK cell treatment, the tumor burden in the inferior lobe of the left lung was significantly reduced (compared to the corresponding image in (a)). (e) Chest CT indicated almost complete absence of lesions in the left lung corresponding to the image shown in (b). (f) Shrinking of lung metastatic lesions was noted.

To evaluate the efficacy of CIK cell treatment, patients underwent regular ultrasonography, chest CT/MRI, or whole-body PET/CT. Unique Patient Number (UPN) 7, who had pulmonary metastasis after radical nephrectomy, showed shrinking of pulmonary lesions and stable disease maintained until the end of followup (Figure 4).

## 4. Discussion

RCC is the most common type of kidney cancer and the third malignancy within urological oncology, accounting for 2-3% of all malignancies and approximately 20−30% of patients with metastatic disease [14], for which the reported median survival is approximately 6 months. Because of the occurrence of spontaneous remission in advanced renal cancer [15], the immune system is thought to play a role in the natural disease course of RCC. Nonspecific cytokine strategies and various forms of immunotherapy, including interleukin-2 (IL-2) and interferon-*α* (IFN-*α*) treatments in association with substances such as 13-*cis*-retinoic acid and/or 5-fluorouracil as monotherapy, are used in the treatment of RCC [16, 17]. Furthermore, cytokine immunotherapy renders an effective survival benefit and has shown biological activity in a number of patients.

Adoptive immunotherapy has now been available for nearly 30 years and holds great promise among potential new approaches for the treatment of solid tumors refractory to conventional therapies [18]. Several conventional adoptive immunotherapies, such as lymphokine-activated killer cells (LAK), tumor-infiltrating lymphocytes (TIL), and anti- CD3 monoclonal antibody-induced killer cells [19–21], have been researched and applied in clinical practice, but their therapeutic efficacy is limited because of their low antitumor activities [22]. LAK cells in combination with IL-2 have been researched extensively and their heterogeneity and capacity to kill both allogeneic and autologous tumors have been demonstrated [23]. TILs represent part of the host immune response to human malignancy and include an abundant population of cells with both cytotoxic and helper functions that are reactive to the autologous tumor [24] in addition to containing antigen-specific and -nonspecific cytotoxic lymphocytes [25]. TILs have shown efficacy in the treatment of patients in terminal stages of cancer. However, despite the success of cell transfer therapy for melanoma, which is regarded as an immunosensitive tumor [26], the clinical efficacy of cell immunotherapy in RCC has been far from being satisfactory [27–29]. Although RCC is an immunosensitive cancer, similar attempts in metastatic RCCs have shown limited success [5, 30–32].

Cytokine-induced killer (CIK) cells are a heterogeneous subset of efficient immune effector cells with potent antitumor activity because of the high proliferation of CD3^+^CD56^+^ cells [33, 34], whose biological features make them attractive targets for adoptive immunotherapy [35, 36]. CIK cell precursors are CD3^+^ T lymphocytes with a naive, CD4CD8 double negative (CD4^−^CD8^−^) phenotype [37]. These cells express T lymphocyte markers and the natural killer cell receptor NKG2D (NK group 2, member D), through which they recognize and kill cells expressing the stress-associated ligands MHC-class-I-polypeptide-related sequences A and B (MIC A and MIC B), which are expressed in the tumor microenvironment and after viral infection [38]. The main functional properties that favorably characterize CIK cells are (1) ex vivo expansion, (2) reduced alloreactivity, and (3) MHC-unrestricted tumor-killing [36]. CIK cells proliferate rapidly in vitro and show stronger antitumor activity, a broader target tumor spectrum, and a lower incidence of adverse effects than other reported antitumor effector cells [8, 10]. The ability to efficiently kill tumor cells is the ultimate requirement for candidate immune effectors for adoptive immunotherapy, and antitumor activity is mainly associated with the CD3^+^CD56^+^ fraction [36]. One of the key processes in the antitumor response is the release of IFN-*γ* and TNF-*α* cytokines by Th1 cells. IFN-*γ* has multiple antitumor effects such as the direct inhibition of tumor growth, blocking of angiogenesis, or stimulation of macrophages [33]. TNF-*α*, another Th1 cytokine produced by activated T cells, induces tumor cell necrosis and enhances the activity of NK and T cells [39].

It was reported that CIK cells migrated to tumor sites by the 7th hour after injection and remained detectable at these sites for an additional 9 days [40, 41]. At the tumor site, CIK cells can exert their cytotoxic activity and control tumor growth. Furthermore, CIK cells regulate and improve the immune function of patients with cancer. Indeed, both autologous and allogeneic CIK cells have been used in phase I/II clinical trials for the treatment of various types of cancer [26]. Schmidt-Wolf et al. [42, 43] described the first clinical trial using CIK cells for the treatment of ten patients with progressive metastatic disease resistant to chemotherapy. These authors demonstrated the feasibility and the low toxicity of this approach and described the case of a patient with follicular lymphoma who developed CR. In this study, the overall objective response rate (ORR) in patients with early renal cell carcinoma who underwent radical nephrectomy and received CIK cell immunotherapy was 80%, which indicates that CIK cells immunotherapy could enhance the prognosis of RCC patients after radical nephrectomy.

In conclusion, CIK cells represent a promising tool among cancer adoptive immunotherapy strategies. Our results indicate the feasibility of the clinical application of CIK cells for the treatment of patients with early RCC after radical nephrectomy. Adoptive immunotherapy with CIK cells represents a safe treatment modality with effective clinical responses. Moreover, CIK cell treatment has resulted in a significant improvement in cell immunological function with an increase in absolute numbers of effector cells without serious adverse events. Their easy and inexpensive ex vivo expansion, along with the MHC-unrestricted tumor killing ability, may overcome some of the problems that have limited the diffusion and clinical translation of other immunotherapy approaches. Despite the small number of patients treated to date, the cell immunological and clinical responses observed are encouraging and warrant further studies of cell adoptive immunotherapy including a larger number of patients and those with a lower tumor load, since patients with minimal disease would probably benefit the most from CIK cell immunotherapy. If confirmed in larger scale studies, these promising results may indicate that CIK cell immunotherapy could be an effective adjunctive therapy for the treatment of RCC.

## Figures and Tables

**Table:** (a)

Case	Age/sex	Nidus	Pathologic stage before Nx (TNM)	Location of metastases	Disease state before CIK cell treatment	CIK cycles	Disease state after CIK cell treatment	Disease state by the end of followup	PFS (month)	OS (month)
UPN 1	69/M	Left	T1N0M0	—	CR	4	CR	CR	31	31
UPN 2	43/M	Right	T1N0M0	—	CR	4	CR	CR	42	42
UPN 3	60/M	Left	T1N0M0	—	CR	4	CR	CR	37	37
UPN 4	53/M	Left	T2N0M1	Lung	PD	8	PR	SD	17	28
UPN 5	61/M	Left	T1N0M0	—	CR	4	CR	CR	40	40
UPN 6	57/M	Right	T2N0M1	Lung	PD	8	PR	SD	38	41
UPN 7	79/F	Left	T2N1M0	Retroperitoneal lymph nodes	PD	8	SD	SD	12	26
UPN 8	50/M	Right	T1N0M0	—	CR	5	CR	CR	40	40
UPN 9	63/M	Right	T2N0M0	—	CR	4	CR	CR	32	32
UPN 10	47/M	Left	T1N0M1	Brain (left temporal lobe)	PD	12	SD	SD	33	33

RCC: renal cell carcinoma; CIK: cytokine-induced killer; TNM: tumornodemetastasis.

**Table:** (b)

Case	Age/sex	Nidus	Pathologic stage before Nx (TNM)	Location of metastases at the beginning of followup	Disease state at the beginning of followup	Treatment protocols	Disease state after treatment	Disease state by the end of followup	PFS (month)	OS (month)
UPN 11*	64/M	Right	T1N0M0	—	CR	Partial left nephrectomy	CR	CR	22	36
UPN 12	45/F	Left	T1N0M0	—	CR	—		CR	32	32
UPN 13	41/M	Left	T1N0M1	Lung	PR	Chemotherapy + targeted therapy	SD	PD	7	30
UPN 14	77/M	Right	T1N1M0	Retroperitoneal lymph nodes	SD	IFN-*α* + IL-2	PR	SD	11	38
UPN 15	27/M	Left	T1N0M0	—	CR	—		CR	40	40
UPN 16	66/M	Left	T1N0M1	Cervical vertebra	PD	Chemotherapy + radiotherapy	SD	PD	12	35
UPN 17	60/M	Right	T2N0M0	—	CR	Chemotherapy	PR	SD	19	36
UPN 18	45/M	Left	T1N0M0	—	CR	—		CR	29	29
UPN 19	65/F	Left	T2N0M1	Right adrenal gland	PR	Chemotherapy	PR	SD	16	32
UPN 20	46/M	Right	T1N0M0	—	CR	—		CR	28	28

*UPN 11: the patient got left renal metastasis during the course of followup and got CR again after partial left nephrectomy till the end of followup.

**Table:** (c)

Factors	Group 1	Group 2	*P*
Karnofsky performance status (KPS) (*n*, %)			
80–100	6	7	—
60–80	4	3	—
Corrected calcium (mmol/L)	2.311 ± 0.100	2.308 ± 0.089	0.926
LDH level (U/L)	180.83 ± 24.659	191.56 ± 23.176	0.893
Hemoglobin level (g/L)	114.86 ± 15.416	120 ± 16.269	0.634
Time from diagnosis to systemic radical nephrectomy (<1 year) (*n*, %)	10 (100%)	10 (100%)	—

Group 1: CIK cells treatment group; group 2: control group.

*P* > 0.05; there are no statistical significant differences between two groups.

**Table 2:** The patients' phenotype of peripheral blood mononuclear cells (PBMCs) before and after cell culture.

Duration of cell culture (days)	CD3^+^ (×10^9^)	CD3^+^CD4^+^ (×10^9^)	CD8^+^ (×10^9^)	CD3^+^CD8^+^ (×10^9^)	CD3^+^CD56^+^ (×10^9^)
0	1.99 ± 0.16	1.07 ± 0.23	1.65 ± 0.17	0.83 ± 0.27	0.19 ± 0.17
13	4.1 ± 0.29*	0.82 ± 0.23	3.78 ± 0.25*	3.34 ± 0.19*	0.63 ± 0.27*

The PBMCs from either day 0, before cell culture, or day 13, after cell culture, were analyzed by flow cytometry for different subtypes of T lymphocyte ($\overset{-}{\chi }\pm S$, %).

*P < 0.01 versus before cell culture.

**Table 3:** Peripheral lymphocyte subsets before and after cytokine-induced killer cell transfusion ($\overset{-}{\chi }\pm S$, %).

	CD3^+^ (%)	CD4^+^ (%)	CD4^+^CD8^+^ (%)
Before transfusion	56.70 ± 5.20	22.91 ± 5.00	1.12 ± 0.25
6–8 days after transfusion	70.50 ± 6.70*	37.80 ± 4.50*	1.82 ± 0.37^∆^
12–14 days after transfusion	67.80 ± 7.50*	32.30 ± 3.40*	1.46 ± 0.36*

*P < 0.01, ^∆^ *P* < 0.01 versus before transfusion.

**Table 4:** Adverse effects and response status.

Adverse reaction	Grade		
	I-II	III-IV	Total
Local reaction	0	0	0 (10)
Fever	1	0	1 (10)
Rash	0	0	0 (10)
Digestive discomfort	0	0	0 (10)
Arthralgia	1	0	1 (10)
Anaphylactoid reaction	0	0	0 (10)
Tumor lysis syndrom	0	0	0 (10)
Laryngeal edema	1	0	1 (10)
Fatigue	3	0	3 (10)
Headache	0	0	0 (10)
Muscular soreness	1	0	1 (10)
